# Mitochondrial DNA DAMPs, Inflammation, and Insulin Sensitivity After Dietary Interventions in Adults with Type 2 Diabetes

**DOI:** 10.3390/nu17203248

**Published:** 2025-10-16

**Authors:** Yenni E. Cedillo, Melissa J. Sammy, Meghan G. Taylor, Cody J. Hanick, Courtney M. Peterson, Barbara A. Gower

**Affiliations:** Department of Nutrition Sciences, University of Alabama at Birmingham, Birmingham, AL 35233, USA; mjsammy@uab.edu (M.J.S.); meghan13@uab.edu (M.G.T.); chanick@uab.edu (C.J.H.); cpeterso@uab.edu (C.M.P.); bgower@uab.edu (B.A.G.)

**Keywords:** mtDNA DAMPs, insulin resistance, insulin sensitivity, inflammation, type 2 diabetes

## Abstract

**Background/Objectives**: Mitochondrial damage is implicated in metabolic dysfunction and may contribute to inflammation and insulin resistance, key features of type 2 diabetes. This study examined the relationship among inflammatory markers, mtDNA DAMPs, and insulin sensitivity/resistance, and evaluated their response to three dietary interventions in type 2 diabetes. **Methods**: Data was pooled from two clinical trials involving adults aged 35 to 75 with type 2 diabetes (*n* = 39). Participants followed one of three 12-week diet interventions aimed at enhancing glucose metabolism without causing weight loss. The sample was 74% female and 64% African American with a mean age of 55.6 ± 7.7 years, and 92.3% (*n* = 36) had overweight/obesity. Participants were assigned to either a carbohydrate-restricted, low-fat, or fruit-rich Mediterranean diet. Primary outcomes included insulin resistance (HOMA-IR), insulin sensitivity (Matsuda index), mtDNA DAMPs (ND1, ND6), pro/anti-inflammatory cytokines (IFN-γ, IL-10, IL-6, IL-8, TNF-α), CRP, and cortisol. Associations among mtDNA DAMPs, inflammation, and insulin sensitivity/resistance were examined using regression analysis **Results**: The carbohydrate-restricted diet led to the greatest improvements in insulin sensitivity (72.7%) and reductions in HOMA-IR (41.3%) (*p* = 0.03). All diets increased mtDNA DAMPs, with most observed in the fruit-rich Mediterranean diet and low-fat diet groups and the smallest in the carbohydrate-restricted group. Total mtDNA DAMPs were associated with lower insulin sensitivity (Matsuda index: β = –0.77; SE = 0.31; *p* = *0*.02), and ND6 mtDNA DAMP levels were associated with greater insulin resistance (HOMA-IR: β = 0.90; SE = 0.40; *p* = 0.03) and lower insulin sensitivity (Matsuda index: β = –0.86; SE = 0.33; *p* = 0.01), independent of BMI and race. Proinflammatory cytokines were associated with increased HOMA-IR (β = 0.45; *p* = 0.007) and reduced Matsuda index (β = –0.43; *p* = 0.009) and moderated effects of mtDNA DAMPs on insulin sensitivity/resistance. **Conclusions**: These findings highlight mtDNA DAMPs in metabolic dysfunction in the context of inflammation.

## 1. Introduction

Type 2 diabetes is the eighth leading cause of death in the United States, with disproportionately higher mortality in the Deep South [1]. In 2021, 11.6% of the U.S. population had diabetes [2]. Type 2 diabetes is a condition strongly associated with excess adiposity, particularly abdominal and intra-abdominal fat, which has been associated with insulin resistance and pancreatic β-cell dysfunction [3].

Chronic inflammation resulting from adipose tissue expansion plays a crucial role in the development of metabolic disorders [4,5,6]. Among the molecular contributors to this inflammation are damage-associated molecular patterns (DAMPs), endogenous molecules released during cellular stress or injury that activate pattern recognition receptors on immune cells, initiating an innate immune response in the absence of pathogens [7]. DAMPs are implicated in the pathogenesis of obesity and type 2 diabetes, particularly through inflammasome activation (e.g., NLRP3) and subsequent release of proinflammatory cytokines [8,9].

Mitochondria have emerged as a key source of DAMPs [8,10,11]. Mitochondrial DAMPs, including mitochondrial DNA (mtDNA) and proteins, are released during cellular stress and bind to pattern recognition receptors such as toll-like receptors (TLRs) and NOD-like receptors (NLRs), triggering the production of proinflammatory cytokines (e.g., TNF-α, interleukins, interferon-γ) and reactive oxygen species (ROS), which are elevated in obesity and cardiometabolic diseases [12,13,14,15]. These mtDNA DAMPs activate innate immune pathways, including TLR9, the NLRP3 inflammasome, and the cyclic GMP–AMP synthase–stimulator of interferon genes (cGAS–STING) pathway, promoting systemic inflammation and impairing insulin secretion. In addition to contributing to β-cell dysfunction, mtDNA DAMPs may impair insulin signaling and promote insulin resistance, as shown in skeletal muscle via TLR9-mediated pathways [16]. Obesity also appears to impair the removal of damaged mitochondria, leading to an increase in circulating mtDNA DAMPs and a shift from anti-inflammatory M2 to pro-inflammatory M1 macrophages, thereby reducing insulin sensitivity. These mtDNA DAMPs also reflect mitochondrial dysfunction that disrupts fatty acid oxidation and glucose homeostasis [13,16,17].

Mitochondria are essential for energy production, metabolism, and cellular signaling. Their dysfunction is associated with low energy expenditure, increased oxidative stress, and chronic diseases [6,14]. Diet is a modifiable factor that influences mitochondrial health. Evidence suggests that total energy intake, antioxidant and phytochemical consumption, and dietary fat content modulate mitochondrial signaling and may influence mtDNA DAMP release [18,19,20,21,22]. Various dietary interventions have shown efficacy in improving obesity and type 2 diabetes outcomes [5,20,23,24]. Reducing mtDNA DAMPs through specific dietary patterns may offer a novel approach to managing metabolic diseases through lifestyle interventions. However, no specific dietary regimen has been shown to target mtDNA DAMPs directly.

Given this gap, the present study aimed to investigate the relationships among pro/anti-inflammatory markers, mtDNA DAMPs, and insulin sensitivity/resistance in adults with type 2 diabetes. Specifically, we examined how these measures changed in response to three dietary interventions: a carbohydrate-restricted diet, a low-fat diet, and a fruit-rich Mediterranean diet. Understanding these mechanisms has significant healthcare relevance, as identifying dietary strategies that mitigate mitochondrial stress and inflammation could inform nutrition approaches and improve the prevention and management of type 2 diabetes. We hypothesized that inflammation and mtDNA DAMP levels would be linked to insulin sensitivity/resistance and that dietary interventions would reduce mtDNA DAMPs and inflammation, which would be accompanied by improved insulin sensitivity.

## 2. Materials and Methods

### 2.1. Study Design and Participants

Data were pooled from two separate 12-week clinical trials in adults with insulin-independent type 2 diabetes living in Alabama. The first was a 12-week randomized trial comparing a carbohydrate-restricted diet with a low-fat diet, designed to examine whether carbohydrate restriction without weight loss improves β-cell function (clinical trial NCT03430310) [25]. The second study was a single-arm, 12-week controlled-feeding trial evaluating the effects of a fruit-rich Mediterranean diet on glycemic control, ectopic fat, and cardiovascular risk factors in weight-stable adults with type 2 diabetes (clinical trial NCT03758742) [26].

Eligibility criteria for each trial are reported in the respective parent studies [25,26]. The aggregated sample included African American and Caucasian American adults aged 20 to 75 years with type 2 diabetes diagnosed within the past 10 years, an HbA1c level less than 9.5%, and a body mass index (BMI) between 20 and 55 (calculated as weight in kilograms divided by height in meters squared). Individuals using insulin or with signs of infection were excluded. In the first clinical trial, medication was discontinued one week before baseline testing. All participants were asked to maintain their usual level of physical activity.

All study procedures were approved by the University of Alabama at Birmingham Institutional Review Board. Written informed consent was obtained from all participants, and compensation was provided. Both studies were conducted in accordance with the Declaration of Helsinki and applicable institutional and federal guidelines.

### 2.2. Diet Intervention Protocols

The first clinical trial compared a carbohydrate-restricted diet (9% carbohydrate, 26% protein, 65% fat) to a low-fat diet (55% carbohydrate, 25% protein, 20% fat). A registered dietitian provided individualized meal plans, grocery lists, and behavioral counseling. Participants received weekly grocery deliveries and prepared their own meals, with energy intake adjusted to maintain weight stability based on weekly self-reported weights [25].

The second trial was a controlled feeding study that tested a fruit-rich Mediterranean diet. During the first four weeks, participants gradually increased their whole fruit intake until they reached 50% of their calorie intake as whole fruit. For the remaining eight weeks of the study, they consumed a fruit-rich Mediterranean-style diet, which provided ~50% of daily calories from whole fruits (~16.4 servings/day) alongside 65% carbohydrates, 12% protein, and 26% fat. Meals were prepared in a metabolic kitchen, and participants consumed only the food provided, except for two break meals per week. Energy intake was adjusted as needed to maintain a stable weight [26].

### 2.3. Measures

#### 2.3.1. mtDNA DAMPs

mtDNA DAMPs were analyzed by the Diabetes Research Center’s Bioanalytical Redox Biology Core. mtDNA DAMPs were analyzed at baseline and week 12 in both studies. mtDNA DAMPs were assessed in cell-free sera from human subjects, as previously described [27,28,29,30]. Briefly, cell-free DNA was extracted from 100 µL of sera using a MagMax™ Cell-Free DNA Isolation Kit (Applied Biosystems), following the manufacturer’s instructions. Cell-free DNA was eluted in a 20 µL volume, and 2 µL was used for the mtDNA DAMPs assay. Sequences within the NADH dehydrogenase subunit 1 (ND1) and NADH dehydrogenase subunit 6 (ND6) regions of the mtDNA were amplified by Real-Time polymerase chain reaction using a StepOne Plus Real-Time PCR system (Applied Biosystems). Copies of mtDNA DAMPs per µL were quantified relative to a standard of known copies (12–50,000). Total mtDNA DAMPs represent the sum of ND1 and ND6.

#### 2.3.2. Oral Glucose Tolerance Testing, Matsuda Index, and HOMA-IR

Both studies conducted an oral glucose tolerance test (OGTT) at baseline and post-intervention. Tests were performed in the morning after a ≥8-h fast. Briefly, participants consumed a 75 g oral load of dextrose, and blood was collected at different time points (8 blood sample collections). The Matsuda index assesses whole-body insulin sensitivity using fasting and postprandial glucose and insulin values collected during an OGTT. The formula for calculating the Matsuda index is: $10,000/\sqrt{\left(G0+I0\right)\times (Gmean\times Imean)}$, where G0 = fasting serum glucose concentration, I0 = fasting serum insulin concentration, Gmean = mean serum glucose concentration during an OGTT, and Imean = mean serum insulin concentration during an OGTT [31]. The homeostasis model assessment of insulin resistance (HOMA-IR) index was calculated using the formula based on fasting glucose and fasting insulin: fasting glucose (mg/dL) × fasting insulin (μU/mL)/405 [32].

#### 2.3.3. Cytokines, Cortisol, and CRP

The UAB Metabolism Core Laboratory conducted laboratory analyses. Cytokines were measured in duplicate using MesoScale Discovery (Rockville, MD, USA) Human V-Plex Proinflammatory Panel I kits. The cytokines measured included IFN-γ (pg/mL), IL-10 (pg/mL), IL-6 (pg/mL), IL-8 (pg/mL), and TNF-α (pg/mL). Cortisol was analyzed using immunofluorescence on the TOSOH A1A-900 immunoassay analyzer (South San Francisco, CA, USA). Assay sensitivity is 0.2 ug/mL, intra-assay CV is 2.56%, and inter-assay CV is 5.19%. *C*-reactive protein (CRP) was assayed with the Stanbio Sirrus using a turbidimetric procedure.

### 2.4. Statistical Analysis

A sensitivity analysis indicated that with 39 participants, the study had 80% power to detect correlations of r ≥ 0.435 at α = 0.05. All statistical analyses were performed using SAS statistical software version 9.4 (SAS Institute Inc., Cary, NC, USA), and graphs were generated in R (RStudio version 2023.06.1). Descriptive statistics (means, standard deviations, and frequencies) were used to summarize baseline characteristics, while within-group changes were reported as mean and SD. All statistical analyses were performed and selected based on data normality characteristics. Within-group changes in pro/anti-inflammatory markers (individual cytokines, CRP, and cortisol) were assessed using the Wilcoxon signed-rank test, stratified by diet intervention group. To identify underlying cytokine profiles, factor analysis with Promax (oblique) rotation was conducted on changes in cytokine concentration levels following the dietary intervention. Pearson correlation analyses were used to assess associations between inflammatory factor scores and changes in insulin sensitivity/resistance. Multiple linear regression models were used to examine associations between cytokine-derived factors, diet intervention group, and changes in insulin sensitivity/resistance, adjusted for race, age, and sex. To assess the association between mtDNA DAMPs (total, ND1, and ND6) and cytokine-derived factors, Spearman correlation analyses were performed. Additional multiple linear regression models adjusted for BMI and race were used to evaluate the influence of mtDNA DAMPs on insulin sensitivity and resistance following dietary intervention. The interaction terms between mtDNA DAMPs and cytokine-derived factors were included to assess potential effects modification. The median of factors was used to graph mtDNA DAMPs according to insulin sensitivity/resistance.

The residuals from all regression models were visually inspected to assess normality, and those with extreme outliers (>2 standard deviations from the mean) were excluded. Logarithmic transformation was applied to skewed variables, including mtDNA DAMPs (total, ND1, and ND6), to meet model assumptions (multiple regression analysis). A two-sided alpha level of 0.05 was considered statistically significant.

## 3. Results

The aggregate sample consisted of 39 adults, including 29 females and 10 males, of whom 25 were African American and 14 were Caucasian American. Participants had a mean age of 55.6 ± 7.7 years and similar baseline BMI (33.5 ± 6.1 kg/m^2^) across groups. About 92% (*n* = 36) of the participants had overweight or obesity. In the second clinical trial, 6 out of 10 participants following a fruit-rich Mediterranean diet decreased their doses of antihyperglycemic medications due to improvements in 24-h glucose levels, while only one participant in the first clinical trial resumed or continued using antihyperglycemic medication.

### 3.1. Insulin Sensitivity/Resistance, Cytokines, and Diet Intervention

As shown in Table 1, HOMA-IR decreased most in the carbohydrate-restricted diet (41.3%), followed by the low-fat (21.0%) and fruit-rich Mediterranean (8.9%) diets. The differences were statistically significant, independent of race (*p* = 0.03). The Matsuda index improved by 72.7% in the carbohydrate-restricted diet, indicating the greatest increase in insulin sensitivity. In Table 2, within-group analyses revealed differential cytokine responses. Following the intervention, the fruit-rich Mediterranean diet saw significant reductions in IFN-γ (*p* = 0.048) and increases in the anti-inflammatory cytokine IL-10 (*p* = *0*.048). The carbohydrate-restricted diet demonstrated a significant decrease in TNF-α levels (*p* = 0.004), while no other within-group changes reached statistical significance across the remaining measures or groups. IL-6, IL-8, and CRP levels remained stable across all diet types.

### 3.2. Cytokine-Derived Factors

Factor analysis with Promax (oblique) rotation identified three correlated latent factors, explaining 63% of the shared variance among changes in cytokines. Factor 1 was characterized by strong loadings on IL-8 (0.87) and TNF-α (0.72), representing an innate, cytokine-driven pro-inflammatory response. Factor 2 was defined by high loadings on IFN-γ (0.70) and IL-6 (0.73), along with moderate loading from TNF-α (0.36), consistent with a Th1-type immune activation and systemic inflammation profile. Factor 3 exhibited strong loadings on IL-10 (0.71) and CRP (0.73), reflecting a regulatory and acute-phase inflammatory response. All final communality estimates exceeded 0.77, indicating a good model fit.

### 3.3. Cytokine-Derived Factors and Insulin Sensitivity/Resistance Change

Pearson correlation analyses were conducted to assess associations between cytokine factors and changes in insulin sensitivity/resistance. Factor 1, representing a pro-inflammatory cytokine response (primarily TNF-α and IL-8), was positively associated with changes in HOMA-IR (*r* = 0.38; *p* = 0.02), indicating that higher levels of inflammation were linked to increased insulin resistance. Factor 2, reflecting IFN–γ–driven Th1 immune activation, was inversely associated with changes in insulin sensitivity (*r* = −0.40; *p* = 0.02). Factor 3, characterized by a CRP-driven acute-phase response, was not significantly associated with any insulin-related outcome. These findings guided the inclusion of Factors 1 and 2 in subsequent multivariable regression models.

### 3.4. Cytokine-Derived Factors, Insulin Sensitivity/Resistance, and Diet Interventions

Multiple linear regression models assessed the associations between inflammatory cytokine factors, dietary interventions, and changes in insulin resistance and sensitivity. The proinflammatory cytokine factor (Factor 1) was significantly associated with increased insulin resistance and reduced insulin sensitivity, reflected by higher HOMA-IR (β = 0.45; *p* = 0.007) and lower Matsuda index scores (β = –0.43; *p* = 0.009), indicating that greater proinflammatory activity was linked to worsening insulin function. Compared with the reference diet, the fruit-rich Mediterranean diet was associated with lower HOMA-IR (β = −0.89; *p* = 0.012), suggesting a potential interaction between diet type and inflammatory status. Factor 2 (IFN-γ–driven immune activation) was not significantly associated with either outcome (*p* > 0.05). Models were adjusted for baseline BMI, race, and sex. Race was significantly associated only in the Matsuda index model (β = 0.99; *p* = 0.004), while duration of diabetes was not a significant predictor and was excluded. Final models explained 71% of the variance in HOMA-IR (R^2^ = 0.71; *p* < 0.001) and 58% of the variance in the Matsuda index (R^2^ = 0.58; *p* = 0.002).

### 3.5. mtDNA DAMPs (ND1 and ND6), Cytokine-Derived Factors, Insulin Sensitivity/Resistance, and Dietary Intervention

As shown in Table 3, the fruit-rich Mediterranean and low-fat diets increased ND1 and ND6 the most (34.6, 36.2, and 47.7, 24.7 units, respectively). The low-carbohydrate group showed the smallest increases in mtDNA DAMPs. At baseline and post-intervention, Spearman correlation analyses were conducted to assess associations between cytokine factors and mtDNA DAMPs (total, ND1, and ND6). At baseline, higher total and ND1 mtDNA DAMPs were positively associated with cortisol (r = 0.35–0.40; *p* < *0*.05) and with the pro-inflammatory cytokine Factor 1 (r = 0.33–0.39; *p* < *0*.05) but inversely associated with Factor 2 (r = −0.34 to –0.41; *p* < *0*.05). Post-intervention, total, ND1, and ND6 mtDNA DAMPs were positively correlated with HOMA-IR (r = 0.33–0.43; *p* < *0*.05) and inversely with the Matsuda index (r = −0.38 to –0.45; *p* < *0*.05), suggesting that elevated mtDNA DAMPs were associated with reduced insulin sensitivity. No significant post-intervention correlations were observed between mtDNA DAMPs and CRP, IL-6, TNF-α, or cortisol.

In multivariable linear regression models adjusted for BMI and race, higher log-transformed post-intervention total mtDNA DAMPs levels (logNDs) were associated with lower insulin sensitivity (Matsuda index: β = −0.77; SE = 0.31; *p* = 0.02), explaining 42% of the variance. ND6 mtDNA DAMPs (logND6) were associated with greater insulin resistance (HOMA-IR: β = 0.90; SE = 0.40; *p* = 0.03) and lower insulin sensitivity (Matsuda index: β = −0.86; SE = 0.33; *p* = 0.01), explaining 64% and 49% of the variance in each outcome, respectively. In contrast, log-transformed ND1 (logND1) was not significantly associated with HOMA-IR (β = 0.06; SE = 0.37; *p* = 0.87) but was inversely associated with the Matsuda index (β = −0.74; SE = 0.28; *p* = 0.01), with models explaining 50% of the variance in HOMA-IR and 43% in the Matsuda index.

**Figure 1:**
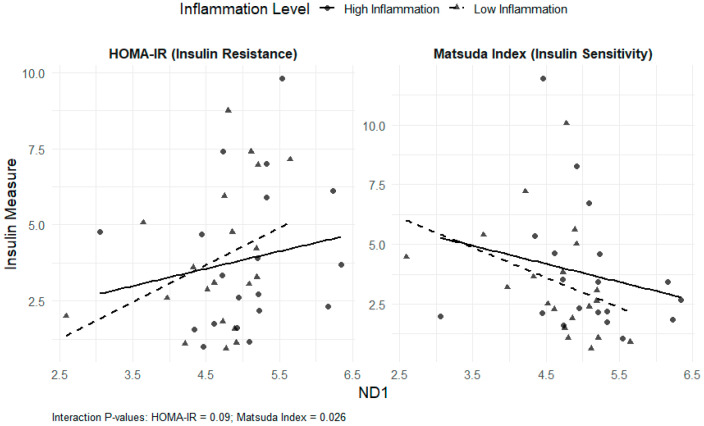
Interaction of ND1 and Inflammation on HOMA-IR and the Matsuda Index.

**Figure 2:**
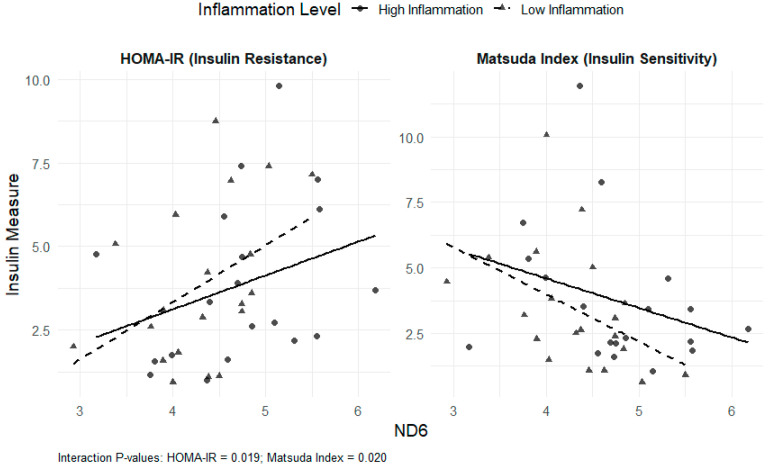
Interaction of ND6 and Inflammation on HOMA-IR and the Matsuda Index.

Interaction models revealed that the associations between mtDNA DAMPs and insulin sensitivity/resistance were influenced by inflammatory status. For total mtDNA DAMPs, significant interactions with Factor 1 were observed for both HOMA-IR (β = −1.75; *p* = 0.02) and the Matsuda index (β = 2.18; *p* = 0.003). For ND6, significant interactions with Factor 1 were observed for both HOMA-IR (β = −0.012; *p* = 0.02) and the Matsuda index (β = 0.010; *p* = 0.02). Similarly, for ND1, a significant interaction with Factor 1 was noted for the Matsuda index (β = 0.009; *p* = 0.03), and a non-significant interaction was observed for HOMA-IR (β = −0.009; *p* = 0.09) (Figure 1 and Figure 2). For Factor 2, the interactions were not statistically significant. These results indicate that the relationships between total mtDNA DAMPs and ND6, as well as insulin resistance or sensitivity, were diminished at higher levels of inflammation.

Multivariable-adjusted regression plots illustrate the relationship between log-transformed ND1 and insulin outcomes, stratified by inflammation level. ND1 was also associated with lower insulin sensitivity in the low-inflammation group, but the interaction was weaker than ND6.

Multivariable-adjusted regression plots show the relationship between log-transformed ND6 and HOMA-IR (left) and Matsuda index (right), stratified by inflammation level (Factor 1 median split). Higher ND6 was associated with greater insulin resistance and lower insulin sensitivity, particularly in the low-inflammation group.

## 4. Discussion

In adults with type 2 diabetes, mtDNA DAMPs were significantly associated with insulin resistance and sensitivity, and these associations were modified by inflammation and diet. At baseline, mtDNA DAMPs were positively associated with serum cortisol concentrations and pro-inflammatory cytokines, suggesting that mitochondrial dysfunction may relate to physiological stress. However, three different dietary interventions that improved insulin sensitivity and/or decreased insulin resistance all increased mtDNA DAMPs. The most pronounced increases in mtDNA DAMPs were observed in the fruit-rich Mediterranean and low-fat diet groups, while the carbohydrate-restricted group showed the smallest changes. Despite a small increase in mtDNA in DAMPs, the carbohydrate-restricted diet led to the greatest improvements in insulin sensitivity, suggesting that short-term mtDAMP changes may not directly reflect metabolic improvements. Elevated proinflammatory activity in this group further underscores the complex interaction among diet, inflammation, and mitochondrial signaling in insulin regulation.

Increased mtDNA DAMPs despite improved insulin sensitivity may reflect adaptive mitochondrial remodeling rather than cellular damage. Dietary interventions that enhance insulin action can stimulate mitochondrial biogenesis and turnover, resulting in a transient increase of mtDNA fragments as part of the renewal process. Improved nutrient signaling and redox balance may also help dampen inflammatory pathways (e.g., TLR9 and NLRP3), thereby reducing the potential adverse effects of DAMP release. These responses likely depend on baseline inflammation and disease control: mtDNA DAMP elevation may amplify immune activation in individuals with a higher inflammatory burden but may indicate beneficial turnover in metabolically stable individuals. Nutrient composition may further shape these effects, as lower-carbohydrate, higher-fat diets produced the smallest DAMP increases and the greatest metabolic improvements. Another possibility is that the predominant fuel oxidized (carbohydrates versus fat) may itself require mitochondrial remodeling to support the shift in substrate utilization, leading to transient changes in mtDNA DAMP release during metabolic adaptation. Inflammation may modify the relationship between mtDNA DAMPs and insulin sensitivity by amplifying immune responses to mitochondrial signals. DAMPs activate pattern recognition receptors (e.g., TLR9, NLRP3), triggering the release of cytokines that can impair insulin signaling. In individuals with elevated inflammation, this response may be intensified, leading to greater metabolic disruption. In contrast, lower inflammation may buffer the impact of mtDNA DAMPs, resulting in a weaker association with insulin resistance. Mechanistically, these findings align with prior evidence linking oxidative stress to mitochondrial dysfunction and insulin resistance. Reactive oxygen species can damage mitochondrial membranes, leading to the release of mtDNA DAMPs, activation of innate immune pathways, and subsequent metabolic impairment [16,33]. The persistent association of mtDNA DAMPs with insulin resistance post-intervention suggests that mitochondrial stress may contribute to impaired insulin signaling independently of traditional inflammatory markers. These results support the hypothesis that mtDNA DAMPs not only reflect cellular stress but may also directly participate in the pathogenesis of metabolic dysregulation in type 2 diabetes.

The observed increase in mtDNA DAMPs in the fruit-rich Mediterranean diet group may reflect transient oxidative stress due to increased mitochondrial biogenesis, elevated dietary fructose intake, or metabolic remodeling [34,35]. These elevations could represent an early physiological adaptation that precedes longer-term improvements in mitochondrial efficiency or insulin sensitivity, as a previous study suggested that consuming a higher-carbohydrate diet triggers a period of metabolic adaptation [36]. Alternatively, higher fructose intake may contribute to oxidative stress and mitochondrial strain in some individuals with type 2 diabetes, suggesting the need for further investigation.

This study has several limitations. The modest sample size (*n* = 39) limits the generalizability of the findings and may reduce the ability to detect smaller effect sizes. Although dietary adherence was carefully monitored, variability in behavioral factors or physiological responses among individuals may have contributed to the observed heterogeneity. The pooling of data from two clinical trials may have also introduced differences in implementation from distinct study protocols or baseline participant characteristics from variations in recruitment and interest in dietary patterns. Changes in antihyperglycemic medication use may also confound some of the findings, since these medications influence insulin response and consequently mtDNA DAMPs levels.

## 5. Conclusions

Higher mtDNA DAMP levels were independently associated with increased insulin resistance and decreased insulin sensitivity in adults with type 2 diabetes. These associations were moderated by systemic inflammation, highlighting the relevance of mtDNA DAMPs as potential biomarkers of metabolic dysfunction in proinflammatory states. Dietary interventions differentially influenced inflammation and insulin sensitivity/resistance. Further research is necessary to assess mitochondrial health and its connection with mtDNA DAMP levels, diet, and insulin sensitivity. Future study design should include a larger sample size with more diverse populations and dietary patterns to reflect variability in mtDNA DAMPs expression. Altering mtDNA DAMPs expression through diet change could offer a new approach to personalized nutrition counseling and long-term chronic disease management.

## Figures and Tables

**Table 1:** Baseline and post-intervention glucose and insulin measurements by diet intervention (*n* = 39).

Characteristics	Diet Intervention (*n*, %)			
	Total	Low-Carbohydrate Diet (*n* = 9)	Low-Fat Diet (*n* = 20)	Fruit-Rich Mediterranean Diet (*n* = 10)
Race				
African American	25(64)	6(67)	14(70)	5(50)
Caucasian American	14(36)	3(33)	6(30)	5(50)
Sex				
Female	29(75)	6(67)	15(75)	8(80)
Male	10(25)	3(33)	5(25)	2(20)
Age	55.6 ± 7.7	54.1 ± 6.3	56.3 ± 8	55.7 ± 8.2
BMI (kg/m^2^) baseline	33.5 ± 6.1	33.9 ± 5.8	34.7 ± 6.2	30.6 ± 5.9
BMI (kg/m^2^) post-intervention	32.4 ± 6.1	32.7 ± 5.5	33.3 ± 6.4	30.4 ± 6.2
Fasting and glucose measures				
Fasting glucose (mg/dL) baseline	131.3 ± 20.3	136.9 ± 19.4	132.6 ± 20.4	123.5 ± 20.6
Fasting glucose (mg/dL) post-intervention	123.6 ± 22.9	121.0 ± 15.2	120.9 ± 19.8	131.5 ± 33.1
Fasting insulin (μIU/mL) baseline	16.6 ± 13.0	14.6 ± 8.6	18.4 ± 15.7	14.7 ± 10.8
Fasting insulin (μIU/mL) post-intervention	13.3 ± 8.5	12.1 ± 8.3	13.7 ± 9.3	13.5 ± 7.8
Matsuda index baseline	3.1 ± 3.3	2.7 ± 1.2	2.6 ± 1.6	4.5 ± 5.9
Matsuda index post-intervention	3.6 ± 2.5	4.7 ± 2.6	3.0 ± 1.9	3.7 ± 3.4
HOMA-IR baseline	4.9 ± 2.9	4.7 ± 1.8	5.3 ± 2.8	4.7 ± 4.2
HOMA-IR post-intervention	3.9 ± 2.4	2.7 ± 1.7	4.2 ± 2.3	4.3 ± 2.9

Categorical data are reported as *n* (%), and quantitative data are reported as means ± SD.

**Table 2:** Inflammation and stress markers by type of diet before and post-intervention (*n* = 39).

Markers	Low-Carbohydrate Diet (*n* = 9)		*p*-Value	Low-Fat Diet (*n* = 20)		*p*-Value	Fruit-Rich Mediterranean Diet (*n* = 10)		*p*-Value
	Baseline	Post-Intervention		Baseline	Post-Intervention		Baseline	Post-Intervention	
IFN-γ (pg/mL)	7.3 ± 7.1	5.2 ± 2.6	0.91	6.3 ± 5.8	6.6 ± 6.6	0.96	6.3 ± 5.7	3.8 ± 2.1	0.048 *
IL-10 (pg/mL)	0.3 ± 0.09	0.2 ± 0.08	0.30	0.3 ± 0.2	0.3 ± 0.3	0.62	0.2 ± 0.1	0.8 ± 1.5	0.048 *
IL-6 (pg/mL)	1.2 ± 0.3	1.3 ± 0.5	0.65	1.4 ± 0.8	1.4 ± 1.5	0.33	1.1 ± 0.7	1.0 ± 0.4	0.49
IL-8 (pg/mL)	9.6 ± 2.9	9.6 ± 3.1	0.82	14.3 ± 12.9	14.7 ± 13.4	0.25	11.9 ± 4.0	12.9 ± 5.7	0.43
TNF-α (pg/mL)	1.7 ± 0.7	1.5 ± 0.6	0.004 *	2.2 ± 0.8	2.0 ± 0.7	0.35	2.3 ± 0.5	2.4 ± 0.5	0.43
CRP (mg/L)	3.1 ± 2.5	3.6 ± 2.9	0.36	3.9 ± 3.7	3.34 ± 3.2	0.21	3.5 ± 2.8	3.5 ± 2.9	0.57
Cortisol (ug/mL)	11.5 ± 3.5	9.7 ± 2.2	0.05	11.1 ± 4.0	11.5 ± 3.1	0.78	12.1 ± 4.2	12.7 ± 3.5	0.56

Data are reported as mean ± SD at baseline and post-intervention. CRP: *C*-reactive protein. *p* values reflect Wilcoxon signed-rank tests comparing pre- and post-change within each diet group. * Significant changes (*p* < 0.05).

**Table 3:** Change in mtDNA DAMPS (Total, ND1, and ND6) by dietary intervention (*n* = 39).

	Time Point	Total mtDNA DAMPs Mean (SD)	ND1 Mean (SD)	ND6 Mean (SD)	Change in ND1 Copies	Change in ND6 Copies
All participants	Baseline	218.2 (133.7)	125.9 (79.6)	92.3 (60.4)	–	–
	Post-intervention	277.3 (202.6)	160.8 (120.0)	116.4 (89.2)	34.9	24.1
Dietary Intervention						
Low-Carbohydrate	Baseline	189.9 (81.3)	110.9 (66.7)	79.0 (35.0)	–	–
	Post-intervention	206.1 (86.1)	117.9 (43.7)	88.2 (53.5)	7.0	9.2
Low-Fat	Baseline	205.6 (118.1)	123.0 (69.9)	82.6 (51.5)	–	–
	Post-intervention	278.0 (197.8)	170.7 (127.3)	107.3 (74.4)	47.7	24.7
Fruit-rich Mediterranean	Baseline	268.9 (189.9)	145.1 (108.7)	123.9 (85.1)	–	–
	Post-intervention	339.8 (273.3)	179.7 (149.8)	160.1 (127.4)	34.6	36.2

Data are reported as mean ± SD. Changes represent absolute mean differences from baseline. Total: ND1 + ND6. ND1: NADH dehydrogenase subunit 1, ND6: NADH dehydrogenase subunit 6 regions of the mtDNA.

## Data Availability

The datasets generated and analyzed during the current study are still in use and not publicly available but are available from the corresponding author upon reasonable request.

## Abbreviations

The following abbreviations are used in this manuscript:

DAMPs	Damage-associated molecular patterns
mtDNA	Mitochondrial DNA
ROS	Reactive oxygen species
BMI	Body mass index
OGTT	Oral glucose tolerance test
HOMA-IR	Homeostasis model assessment of insulin resistance
CRP	*C*-reactive protein
NADH	Nicotinamide adenine dinucleotide + hydrogen
